# ALS-Associated E478G Mutation in Human OPTN (Optineurin) Promotes Inflammation and Induces Neuronal Cell Death

**DOI:** 10.3389/fimmu.2018.02647

**Published:** 2018-11-14

**Authors:** Zhengzhao Liu, Hongming Li, Chungu Hong, Menglu Chen, Tao Yue, Chunyuan Chen, Zhenxing Wang, Qing You, Chuanyin Li, Qinjie Weng, Hui Xie, Ronggui Hu

**Affiliations:** ^1^Movement System Injury and Repair Research Center, Xiangya Hospital, Central South University, Changsha, China; ^2^Hunan Key Laboratory of Organ Injury, Aging and Regenerative Medicine, Changsha, China; ^3^Department of Sports Medicine, Xiangya Hospital, Central South University, Changsha, China; ^4^Shenzhen Second People's Hospital, First Affiliated Hospital of Shenzhen University, Shenzhen, China; ^5^Department of Orthopedics, Xiangya Hospital, Central South University, Changsha, China; ^6^State Key Laboratory of Molecular Biology, CAS Center for Excellence in Molecular Cell Science, Innovation Center for Cell Signaling Network, Shanghai Institute of Biochemistry and Cell Biology, University of Chinese Academy of Sciences, Shanghai, China; ^7^Department of life science, Shanghai Tech University, Shanghai, China; ^8^Institute of Pharmacology and Toxicology, Zhejiang Province Key Laboratory of Anti-Cancer Drug Research, College of Pharmaceutical Sciences, Center for Drug Safety Evaluation and Research, Zhejiang University, Hangzhou, China; ^9^National Clinical Research Center for Geriatric Disorders, Changsha, China

**Keywords:** optineurin, ALS, inflammation, cell death, cytokines

## Abstract

Amyotrophic Lateral Sclerosis (ALS) is a group of neurodegenerative disorders that featured with the death of motor neurons, which leads to loss of voluntary control on muscles. The etiologies vary among different subtypes of ALS, and no effective management or medication could be provided to the patients, with the underlying mechanisms incompletely understood yet. Mutations in human *Optn* (Optineurin), particularly E478G, have been found in many ALS patients. In this work, we report that NF-κB activity was increased in *Optn* knockout (*Optn*^−/−^) MEF (mouse embryonic fibroblast) cells expressing OPTN of different ALS-associated mutants especially E478G. Inflammation was significantly activated in mice infected with lenti-virus that allowed overexpression of *OPTN*^E478G^ mutation in the motor cortex, with marked increase in the secretion of pro-inflammatory cytokines as well as neuronal cell death. Our work with both cell and animal models strongly suggested that anti-inflammation treatment could represent a powerful strategy to intervene into disease progression in ALS patients who possess the distinctive mutations in *OPTN* gene.

## Introduction

ALS (amyotrophic lateral sclerosis) is a neurodegenerative disease that is characterized by the progressive degeneration of motor neurons both in the brain and spinal cord (1). About 10% of ALS cases are transmitted in families. The first genetic mutations affected gene *SOD1* found to associate with 1–3% of sporadic ALS, while 5% or more are caused by intronic expansion in C9orf72 (2–4). *OPTN* (*Optineurin*) has also been identified in patients with family or sporadic ALS. But validating the causality of specific variants remains challenging and yet unachieved (1).

OPTN is an autophagy receptor that selectively binds to cargos marked for degradation and delivers them to autophagosomes (5, 6). Previous studies had established a strong link between ALS and mutations and polymorphisms of OPTN (7), with three types of mutations in *OPTN* (a homozygous deletion of exon 5, a homozygous Q398X nonsense mutation, and a heterozygous E478G missense mutation) identified in Japanese ALS patients (8, 9). More mutations were discovered subsequently in cohorts of patients of various descents, such as Q165X, Q454E (10, 11); R96L, 382_383insAG (12, 13); K59N, A481V (14); T282P, Q314L, K557T, G23X (15). Mutations in *OPTN*, particularly E50K, were also found in 16.7% of hereditary POAG families (primary open-angle glaucoma), (16). Comparative studies of these mutants might shed light on the tissue-specific functional consequences of *OPTN* mutations and the mechanisms of disease as well.

Turturro et al. examined the effects of some ALS-associated *OPTN* mutations or deletions on foci formation, Golgi integrity, protein trafficking and revealed that Q398X and 382_383insAG were abnormal among Golgi fragmentation, transferrin uptake and cell apoptosis (17). There remained a gap to understand whether and how this mechanism might contribute to the pathology of the disease, and much more work needs to be done to uncover the direct causality of the ALS-associated genetic abnormality in *OPTN*.

On the other hand, there has emerged a body of evidences that have established OPTN as a novel potent regulator of cellular inflammatory pathways. *Optn* suppression was shown to cause neuronal cell death via NF-κB pathway (18). OPTN was identified as a negative regulator of TNF-a induced NF-κB activation (19, 20). Moreover, OPTN inhibited NF-κB activation by competing with NEMO in order to bind ubiqitinated RIP1 (receptor-interacting protein 1) (21). Once bound to RIP1, OPTN directly interacted with cylindromatosis (CYLD) to mediate deubiquitination of RIP1 by CYLD and thereby blocked downstream of NF-κB signaling pathway (19). Overexpression of OPTN was shown to down-regulate IL-1β, IRAK1 (Interleukin-1 receptor-associated kinase 1), and LPS induced NF-κB activation by preventing polyubiquitination of TRAF6. (22). However, previous studies from four independent groups (23–26) revealed that optineurin was dispensable for NF-κB activation while necessary for IRF3 activation using the OPTN^D477N^, OPTN^470T^, OPTN^Δ157^ and *Optn*^−/−^ mouse model (5, 23–26). Since OPTN may regulates the function of motor neurons by regulating NF-κB signaling pathway, it is compelling to ask how those factors work in mouse models relevant for ALS. Determining the extent to which OPTN plays a role in ALS will provide novel trains of thought about ALS pathogenesis, and might come out with effect treatment to this neurodegenerative disease.

Herein, we examined the role of E478G mutation upon NF-κB activation and found that E478G substitution or those mutations in UBA (ubiquitin binding associated domain) could overtly abolished the inhibitory effect of OPTN on NF-κB activation, as reported previously. Our data further suggested that IL-1β was significantly up-regulated in OPTN^E478G^ overexpressed cells and mice. Therefore, our work has identified IL-1β as the key effector of the ALS-associated OPTN^E478G^ mutation in neuroinflammation and neuronal cell death. Given that the activated neuroinflammation could serve as a major driving force in etiology of ALS, combating neuroinflammation might represent a powerful strategy to bring beneficial outcomes in ALS therapy.

## Results

### ALS-associated E478G mutation abolished the inhibitory effect of OPTN on NF-κB activation in *Optn^−/−^* MEF cells

**Figure 1:**
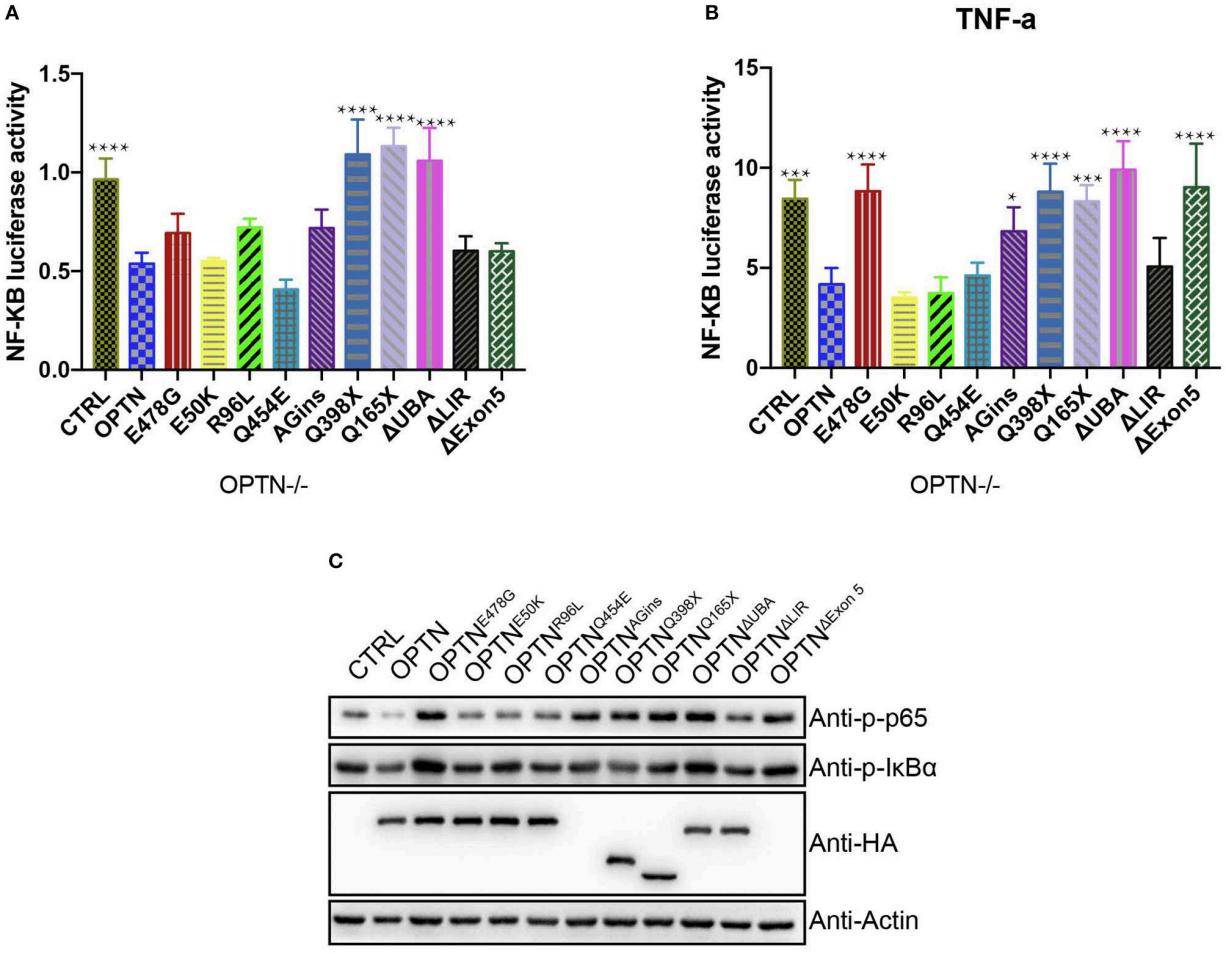
OPTN^E478G^ abolished NF-kB activation-inhibiting effect in *Optn*^−/−^ MEF cells. **(A)** NF-κB activity was measured by dual luciferase reporter assay after transfecting *Optn*^−/−^ MEF cells with NF-κB reporter and the mutants of *OPTN* plasmid as indicated. Data were presented as mean ± SEM, *n* = 4. Data analysis was performed by comparing each group to OPTN group using one way ANOVA and Dunnett's multiple comparison test, ^****^p ≤ 0.0001, without asterisks means no significance *p* > 0.05. **(B)** NF-κB activity was measured by dual luciferase reporter assay after transfecting *Optn*^−/−^ MEF cells with NF-κB reporter and the mutants of *OPTN* plasmid as indicated. 24 h after transfection, cells were treated with 20 ng/ml TNF-a for 4 h before harvest. Data were presented as mean ± SEM, *n* = 4. Data analysis was performed by comparing each group to OPTN group using one way ANOVA and Dunnett's multiple comparison test, ^****^p ≤ 0.0001, ^***^p ≤ 0.001, ^*^p ≤ 0.05, without asterisks means no significance *p* > 0.05. **(C)** NF-κB activity was examined by bloting p-IκBα and p-P65, GAPDH as a loading control. Overexpression of the mutants in *Optn*^−/−^ MEF cells were confirmed by HA immunoblotting. Q398X or Q165X generates a premature stop codon and truncates OPTN to 397aa or 164aa in the length respectively. 382_383AGins (also known as 691_692AGins) introduces a premature stop codon in exon 6 and generates 148aa fragment of OPTN. ΔExon5 lead to a frame shift and generates 58aa fragment of OPTN.

Although multiple mutations in OPTN and its deletion had been found in ALS patients, it remained undetermined the exact causality of the disease. While other work has suggested potential association between the activated neuroinflammation and motor neuronal cell death. To further assess the validity of such association, we set out to assess the effect of different ALS-related OPTN mutants systematically on the functionality of cellular inflammatory pathways. Wild-type OPTN or the ALS-associated mutants were then cloned into pCDH lenti-virus vector with a HA tag fusion at their C- terminus for expression in *Optn*^−/−^ MEF cells. *Optn*^−/−^ MEF cells expressed each mutation properly (Figure 1C). The cloned mutants include OPTN^E478G^, OPTN^R96L^, OPTN^Q454E^, OPTN^382_383AGins^, OPTN^Q398X^, OPTN^Q165X^, OPTN^ΔExon5^ along with OPTN^E50K^, which was commonly mutated in open angle glaucoma, OPTN^Δ*UBA*^ with a deletion of ubiquitin binding domain(474-479aa), OPTN^Δ*LIR*^ with a deletion of LC3 interaction region(169-184aa).

First, luciferase reporter assays were performed to systematically compare the activities of NF-κB pathway in *Optn-/-* MEF cells (Figures 1A,B). The expression of wild-type *OPTN* was found to potently suppress the activity of NF-κB pathway, with or without TNF-a treatment (20 ng/ml), suggesting an anti-inflammatory function of OPTN. We also chose OPTN^E50K^ as a control to evaluate the NF-κB activity, as Akizuki et al. found that OPTN^E50K^ suppressed NF-κB signaling pathway as the wild-type did in Neuro2a cells (18). OPTN^E50K^ also inhibit NF-κB activity in *Optn*^−/−^ MEF cells and 293T cells (Figures S1A,B). However, the activity of NF-κB pathway was found to be much higher in *Optn*^−/−^ MEF cells expressing other ALS-associated OPTN mutants. Namely, OPTN^AGins^, OPTN^Q398X^, OPTN^Q165X^, OPTN^ΔExon5^, OPTN^ΔUBA^, which commonly lost the C-terminal ubiquitin binding domain, except the OPTN^Δ*LIR*^. Among them, OPTN^E478G^ was one of those which has compromised inhibitory effect on NF-κB activity, with or without TNF-a treatment. Such effect seemed to be independent of cell types. similar results were found in *Optn*^−/−^ MEF cells and 293T cells (Figures S1A,B). Consistently, in immunoblotting assays to examine the levels of endogenous p-IκBα and p-P65 protein level, p-IκBα and p-P65 were increased markedly in OPTN^E478G^ group in comparing to those expressed wild-type OPTN. Interestingly, similar phenomena were also found in OPTN^AGins^, OPTN^Q398X^, OPTN^Q165X^, OPTN^ΔExon5^, OPTN^ΔUBA^. These results strongly suggested that the ubiquitin binding domain in OPTN might be essential for its suppressing effect on the activation of NF-κB pathway.

### Secretion of pro-inflammation cytokines was up-regulated in OPTN^E478G^ transfected cells

**Figure 2:**
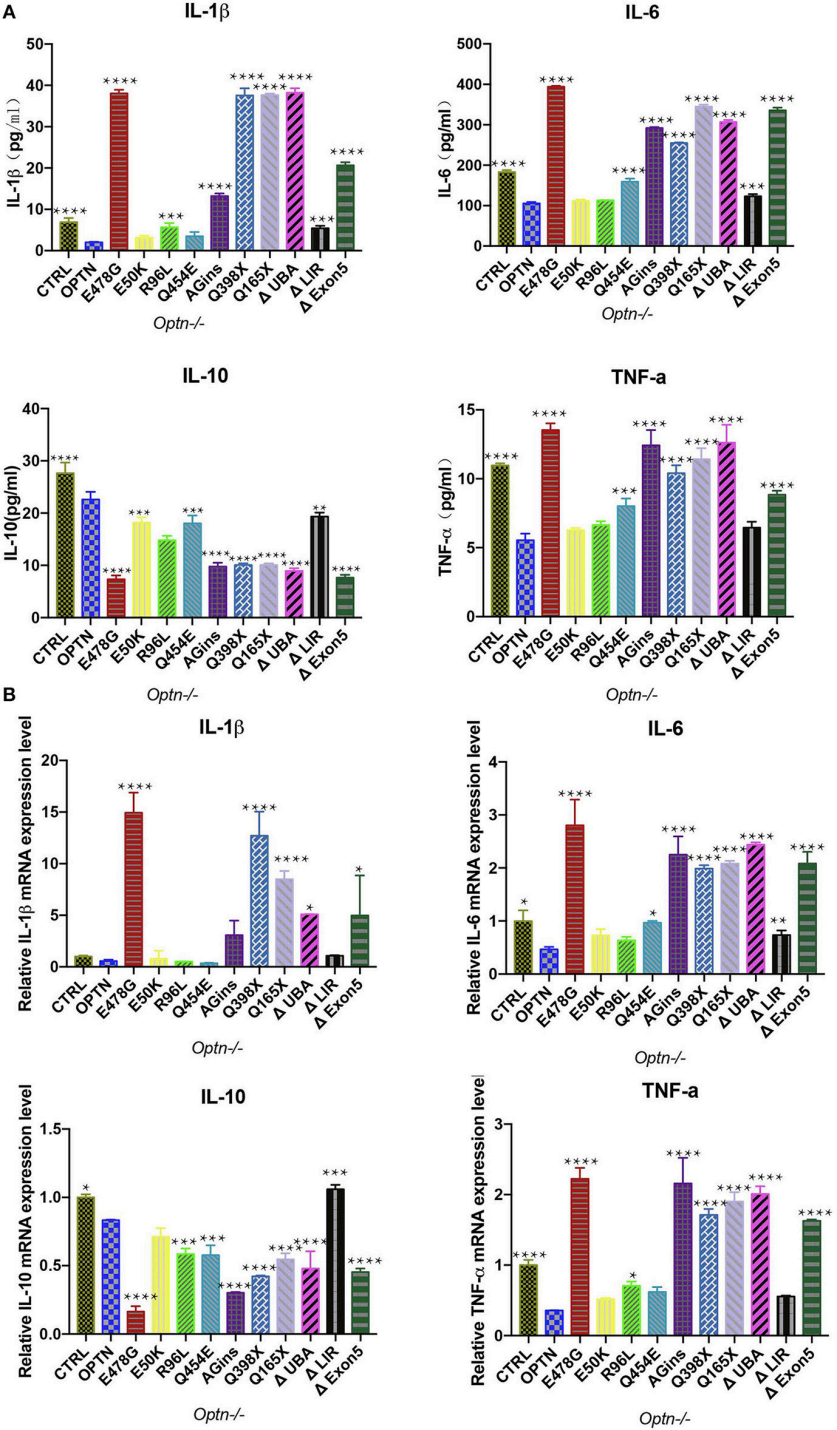
Secretion of cytokines was analyzed in *Optn*^−/−^ MEF cells transfected with ALS-associated *OPTN* mutants. **(A)** Cytokines (IL-1β, IL-6, IL-10, TNF-a) in the supernatants of the cell culture medium were analyzed by enzyme-linked immunosorbent assay (ELISA). Data were presented as mean ± SEM, *n* = 3. Data analysis was performed by comparing each group to OPTN group using one way ANOVA and Dunnett's multiple comparison test, ^****^p ≤ 0.0001, ^***^p ≤ 0.001, without asterisks means no significance *p* > 0.05. **(B)** Expression of cytokines (IL-1β, IL-6, IL-10, TNF-a) were measured by quantitative real-time PCR. Data were presented as mean ± SEM, *n* = 3. Data analysis was performed by comparing each group to OPTN group using one way ANOVA and Dunnett's multiple comparison test, ^****^p ≤ 0.0001, ^***^p ≤ 0.001,^**^p ≤ 0.01,^*^p ≤ 0.05, without asterisks means no significance *p* > 0.05.

To further pinpoint which inflammatory cytokines might be up-regulated upon expression of OPTN^E478G^, expression and secretion of these cytokines were examined, using quantitative real-time PCR (qPCR) and ELISA, respectively. Interestingly, in comparison with that of the *Optn*^−/−^ MEF cells transfecting with wild-type *OPTN* and ALS-associated *OPTN* mutants (Figures 2A,B). IL-1β, IL-6, and TNF-α but not IL-10, were found to be elevated in culture media for the cells expressing the *OPTN* mutaions. Specifically, both expression and secretion of IL-1β, IL-6, or TNF-α were significantly increased with cells expressing OPTN^E478G^, OPTN^AGins^, OPTN^Q398X^, OPTN^Q165X^, OPTN^ΔExon5^, OPTN^ΔUBA^, when compared to the OPTN; however, opposite effects were observed with IL-10. It is interesting to note again that all the tested mutants, manifesting the above effect, were all those bearing mutants disrupting the UBA domain of OPTN, e.g., OPTN^E478G^, OPTN^AGins^, OPTN^Q398X^, OPTN^Q165X^, OPTN^ΔExon5^, OPTN^Δ*UBA*^. These results strongly suggested again that the C-terminal UBA domain of OPTN was essential for the inflammation-suppressing effect of OPTN. The similar phenomena were also true in 293T cells (Figures S2A,B). It was really remarkable that, with the cells expressing OPTN^E478G^, transcription of IL-1β was up-regulated 26-fold in company with 19-fold more secretion, compared to those cells expressing wild-type OPTN. The OPTN^E478G^ was thus selected for all the subsequent *in vivo* studies.

### Infection of OPTN^E478G^ virus caused severe neuroinflammation in mouse motor cortex

**Figure 3:**
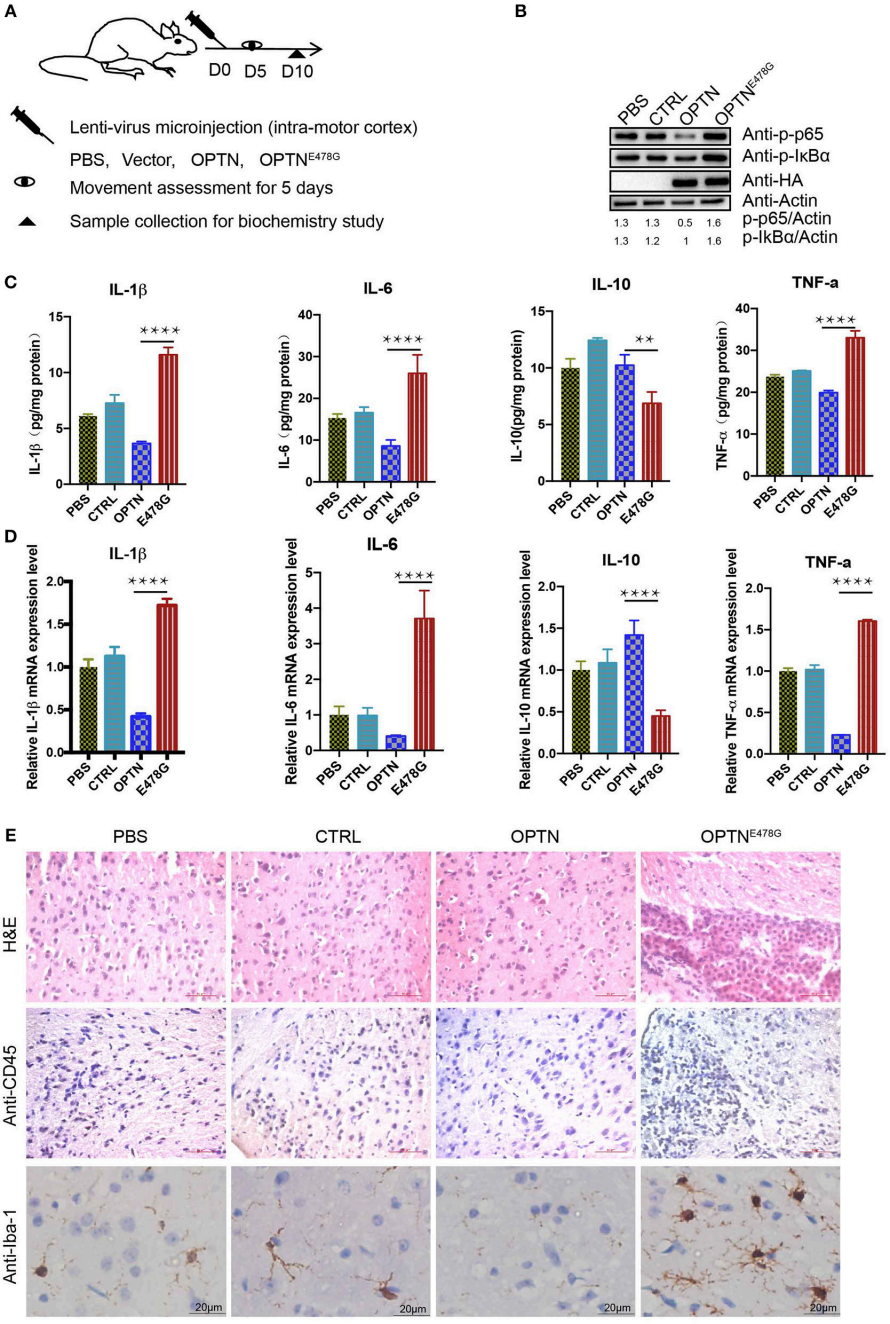
OPTN^E478G^ abolished NF-κB activation-inhibiting effect and had severe inflammation. **(A)** Diagram of the treatment of the mice for subsequent analysis. Assessments of movements started 5 days after lenti-virus microinjection. The assessments were performed for 5 consecutive days. **(B)** NF-κB activity was examined by bloting p-IκBα and p-P65 from the brain tissues following intra-motor cortex injections, GAPDH as a loading control, Overexpression of HA-tagged OPTN WT and E478G in the brain tissues were confirmed by HA immunoblotting. **(C)** Cytokines (IL-1β, IL-6, IL-10, TNF-a) in the brain tissue following intra-motor cortex injections were analyzed by ELISA. Data were presented as mean ± SEM, *n* = 3. Data analysis was performed by comparing E478G group to OPTN group using one way ANOVA and Dunnett's multiple comparison test, ^****^p ≤ 0.0001, ^**^p ≤ 0.01. **(D)** Cytokines (IL-1β, IL-6, IL-10, TNF-a) in the brain following intra-motor cortex injections were measured by quantitative real-time PCR. Data were present in mean ± SEM, *n* = 3. Data analysis was performed by comparing E478G group to OPTN group using one way ANOVA and Dunnett's multiple comparison test, ^****^p ≤ 0.0001. **(E)** Lymph cells was examined by hematoxylin and eosin and CD45 staining (upper and middle panel), Iba-1 stained the microglia cells in the injected motor cortex region (lower panel). Scale bar, 50 μm. Statistic data were presented in Figures S3A,B.

To further investigate the potential effect of the OPTN^E478G^ mutant on neuroinflammation *in vivo*, intra-motor cortexes microinjection was performed (Figure 3A). Mice were grouped as injected with PBS, the control viral vector, virus encoding wild-type OPTN or OPTN^E478G^. 10 days after the microinjection, samples from the brain were harvested to examine the levels of endogenous p-IκBα and p-P65, which clearly indicated the activation of NF-κB pathway upon injection of OPTN^E478G^. ELISA and qPCR assays revealed that IL-1β, IL-6, or TNF-a were significantly upregulated at both mRNA and protein levels in the OPTN^E478G^ group, when compared to that of control group or that injected with lenti-virus encoding wild-type OPTN; On the contrary, IL-10 was decreased in OPTN^E478G^ group for the same comparison (Figures 3B–D). These data, in line with those from the in vitro cell-based analysis, clearly indicated an activated inflammation state in the mouse brains expressing OPTN^E478G^. Results from the histological staining and immunostaining assays further revealed that there were indeed markedly more lymphocytes and microglia with higher expression of CD45, a prominent marker shared by monocyte, macrophages, lymphocyte, and microglia, in the brain tissue of mice infected with OPTN^E478G^ (Figure 3E, Figure S3A). Positive staining of Iba-1, a microglia-specific marker (27), was also significantly increased in the same comparison (Figure 3E; Figure S3B).

### OPTN^E478G^ induced nerve cell death and abnormal motility

**Figure 4:**
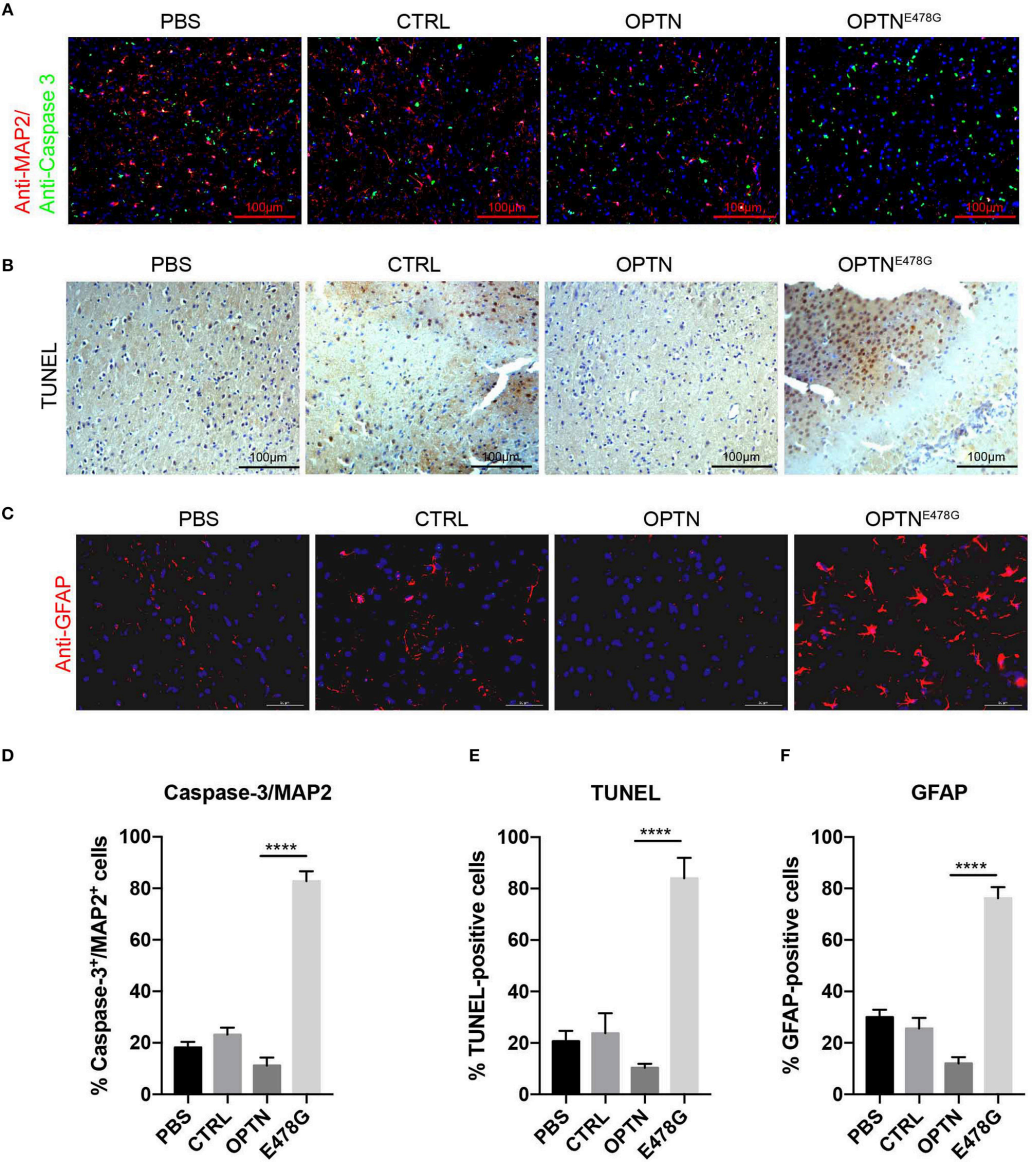
Infection of OPTN^E478G^ virus increased neuronal cell death and astrogliosis. **(A)** Co-immunofluorescent staining of Caspase-3 and MAP2 in mice brain after intra-motor cortex microinjection with lenti-virus bearing control vector, wild-type OPTN or OPTN^E478G^. DAPI staining to show the nucleus in blue, MAP2 was shown in red, Caspase-3 was shown in green. **(B)** Detection of cell death in mice brain section by TUNEL assay after intra-motor cortex microinjection. **(C)** Immunohistochemically staining to analyze astrocytes after intra-motor cortex microinjection. DAPI staining to show the nucleus in blue, GFAP was shown in red. **(D)** Percentage of Caspase-3^+^/ MAP2^+^ cells. Values were presented as mean ± SEM, *n* = 6. Data analysis was performed by comparing E478G group to OPTN group using unpaired *t-*test, ^****^p < 0.0001. **(E)** Quantification of TUNEL-positive cells. Values were presented as mean ± SEM, *n* = 6. Data analysis was performed by comparing E478G group to OPTN group using unpaired *t-*test, ^****^p < 0.0001. **(F)** Quantification of GFAP-positive cells. Values were presented as mean ± SEM, *n* = 6. Data analysis was performed by comparing E478G group to OPTN group using unpaired *t-*test, ^****^p < 0.0001.

Having observed activated inflammation in mouse brains overexpressing OPTN^E478G^, neuronal cell death was further assayed by the co-immunofluorescent staining with anti-Caspase-3 and anti-MAP2 antibodies. When compared to the OPTN wild-type group, the ratio of Caspase-3 positive cells to MAP2 positive cells was dramatically increased in E478G mutant group, suggesting elevated death in nerve cells, especially neuron cells (Figures 4A,D). TUNEL assays further confirmed increased nerve cell death in mouse brains of the E478G group, when compared to those injected with virus only or those encoding wild-type OPTN (Figures 4B,E). Previous work established that reactive astrogliosis develops at the onset of symptoms, after the loss of character of motor neuron by the hypertrophy of cellular processes, and along with the up-regulation of the markers GFAP and Vimentin, which is accompanied by increased microglia (28–30). Immunofluorescence staining of brain tissue after intra-motor cortex microinjection with anti-GFAP antibody found that GFAP was substantially enhanced, indicating the development of a strong reactive astrogliosis around the degenerating motor neurons (Figures 4C,F).

**Figure 5:**
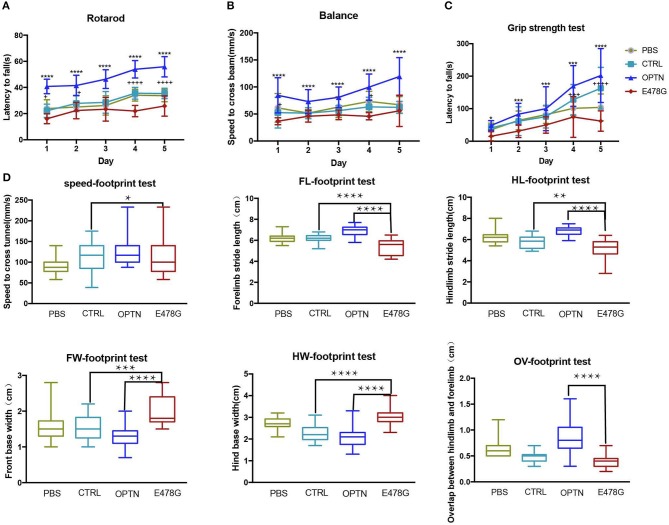
OPTN^E478G^ virus-infected mice had impaired motility. **(A)** Movement of mice was evaluated by rotarod test for 5 consecutive days (*n* ≥ 6). Values were presented as mean ± SEM, (*n* ≥ 6, 3 repeats for each mouse). Data were analyzed using two way ANOVA and Dunnett's multiple comparison test, ^*^ means significance between E478G group and OPTN group, + means significance between E478G group and CTRL group. ^****^,^++++^*p* ≤ 0.0001; ^+^*p* ≤ 0.05. **(B)** Movement of mice was evaluated by Grip strength test for 5 consecutive days (*n* ≥ 6). Values were presented as mean ± SEM, (*n* ≥ 6, 3 repeats for each mouse). Data were analyzed using two way ANOVA and Dunnett's multiple comparison test, ^*^ means significance between E478G group and OPTN group, + means significance between E478G group and CTRL group. ^****^p ≤ 0.0001, ^+^*p* ≤ 0.05. **(C)** Movement of mice was evaluated by Balance beam test for 5 consecutive days (*n* ≥ 6). Values were presented as mean ± SEM, (*n* ≥ 6, 3 repeats for each mouse). Data were analyzed using two way ANOVA and Dunnett's multiple comparison test, ^*^ means significance between E478G group and OPTN group, + means significance between E478G group and CTRL group. ^****^,^++++^*p* ≤ 0.0001; ^***^,^+++^*p* ≤ 0.001; ^*^p ≤ 0.05. **(D)** Movement of mice was evaluated by footprint test. Whisker-boxplots that showed various parameters measured in footprint test after intra-motor cortex microinjection (*n* ≥ 6). Values were presented based on the median and quartiles, *n* ≥ 18 (3 repeats for each mouse). Data were analyzed using one way ANOVA and Dunnett's multiple comparison test. FW, front base width; HW, hind base width; FL, forelimb stride length; HL, hindlimb stride length; OV, overlap between hindlimb and forelimb (Also see Figure S3C). ^****^p ≤ 0.0001, ^**^p ≤ 0.01, ^*^p ≤ 0.05.

In light of the symptomatic impairment in locomotivity of ALS patients, mice infected with the OPTN^E478G^ lenti-virus along with the other groups of mice were subjected to series of motility tests, which including (1) the accelerating-rotarod and balance beam tests in order to examine the motor coordination and balance of the mice, (2) the additional grip strength test to determine muscle tension, (3) and the footprint test to monitor the mouse gaits. As shown in Figure 5, the OPTN^E478G^ mice fell off the rotarod more quickly than both control and OPTN group. The grip strength of this group was also significantly weaker and the speed to cross the balance beam was much lower. In footprint test, OPTN^E478G^ mice manifested shorter stride length and wider base width than the control mice or those infected with wild-type OPTN. To summarize, it appeared that the ALS-associated E478G mutation in OPTN could indeed cause substantial impairment in mouse locomotivity, which was most likely due to the more neuronal cell death that OPTN^E478G^ caused in the brains.

## Discussion

Patients of Amyotrophic Lateral Sclerosis (ALS) gradually lose motor ability and self-caring ability. There are yet very limited number of options for ALS managements and treatments. With only 2 drugs, Riluzole, and Edaravone, approved for ALS treatments that provide a mild improvement in survival (31). It is thus impelling to find a symptom-based management that could promote survival and improve the life quality of the patients. Mutations in *OPTN* gene have been found to associate with ALS across the race and continents. Genetic variations in OPTN and TBK1 were estimated to account for or contribute to 1.3% of cases in whole exome sequencing of 2,874 ALS patients (32), comparable to the contributions for those found in many other genes such as SOD1, TDP43, or C9ORF72 (33). So far OPTN has been known to partake in multiple signaling pathways, in which it functioned as a autophagy receptor, regulating vesicle trafficking, and modulating NF-κB activity, with yet unresolved controversy (24, 26). This controversy is especially focused on whether it can inhibit NF-κB activity, since cell-based experiments show strong inhibiting effect whereas almost non-effective in the animal model studies (5, 18–20, 23–26).

In this work, we introduced OPTN, wild-type or ALS-associated mutants, into *Optn*^−/−^MEF cells, and systematically assessed their potential effects on the activity of NF-κB pathway. It was clear that ALS-related mutations in *OPTN* abolished its inhibitory effect on the activity of NF-κB pathway. Similar observations were obtained with all OPTN mutants that commonly had their C-terminal UBA domain missed. Activation of NF-κB by the mutants may not be a dominant negative effect since we re-introduced the OPTN mutants into *Optn*^−/−^ MEF cells that were totally absent from endogenous OPTN protein. Our data deviated from those by the referenced 4 groups, who used OPTN^D477N^, OPTN^470T^, OPTN^Δ157^, and *Optn*^−/−^ mouse model (5, 23–26). Slowicka et al. surveyed the NF-κB signaling pathway by blotting p-IκBα. They did not see increase in TNF-a treatment but increased in poly (I:C) treatment when compared the WT and KO MEF cells (26). But in our case, we detect p-IκBα was decreased when re-expression *OPTN* in *Optn*^−/−^ MEF cells. It is not controversial with results from Slowicka et al. It is probably that the endogenous OPTN level is not sufficient to elicit a significant p-IκBα decrease when compared with OPTN level in overexpressed *Optn*^−/−^ MEF cells. Myruyama et al. had also reported this inhibition effect on NF-kB signaling pathway (8). Virus carrying *OPTN* mutants should be microinjected into the *Optn*^−/−^ mice instead of wild-type mice to further confirm our results.

Interestingly, OPTN^E478G^ expression was found to elicit the strongest induction of IL-1β transcription (26-fold increase) and secretion (19-fold higher), compared to wild-type OPTN (Figure 2). Although MEF cell is a quite remote cell type from neurons, but we used this cell type as a *Optn* knockout cell to evaluated the effects of ALS-associated mutants in NF-κB signaling. Similar results were also obtained in 293T cell lines and viral expression assays in *in vivo* experiment. Increased IL-1β production and more TUNEL positive cells in Optn knockout mice were also found by Ito et al. (34). We next used *in vivo* mice model to confirm that OPTN^E478G^ intra-motor cortex injection did affect IL-1β secretion in the brain tissue. OPTN^E478G^ was found to significantly elevate inflammation in the brains with concomitant increase in astrocyte, microglia and the neuronal cell death (Figures 3, 4). Animal behavior tests further indicated that the motility of the mice was also severely impaired by OPTN^E478G^ (Figure 5). Notably, our data further suggested that the neutralization of the IL-1β with anti-IL-1β antibody could ameliorate OPTN^E478G^- caused damage in mouse motor ability (data not shown).

Collectively, our data indicated that the UBA domain of OPTN is important for the NF-κB inhibition effect in a OPTN^E478G^-virus mediated overexpressing mouse model. This may due to the aberrant binding to the ubiquitinated RIP1 and recruiting the CYLD to deubiquitinated RIP1 by the mutation in UBA domain of OPTN, resulting in the inflammation and secretion of pro-inflammation cytokines especially IL-1β, which was consistent with the previously reported findings (22). We hypothesize that the E478G mutation in OPTN might significantly change the protein structure, at least locally, thus disrupting its ubiquitin binding ability as suggested before (35). The inflammation status with increased astrocytes, microglia cells and nerve cell death, would reflect on the losing motor ability gradually. It is also valuable to assay whether anti-IL-1β treatment could protect the nerve cells through ameliorating the inflammation response, thus promoting the survival of nerve cells. Our work indicated that the ALS-associated mutants of OPTN promoted death in the nerve cells, most likely neurons as the consequence of activation of NF-κB pathways. Anti-IL-1β treatment may exert benefit effect on this sub-group of ALS patients who bear the herein studied OPTN mutations.

We have previously demonstrated that ubiquitylation of OPTN by tumor-suppressing E3 ubiquitin ligase, HACE1, promotes the formation of autophagy receptor complex and activates autophagy (36). It is intriguing to explore whether and how post-translational modifications may be employed to purposefully manipulate the functionality of ALS-causing OPTN mutations for clinical benefits.

## Materials and methods

### Ethics statement

All procedures for animal uses were in strict accordance with the guidelines of animal welfare set by the World Organization for Animal Health and approved by the Institutional Animal Care and Use Committee of Shanghai Institute of Biochemistry and Cell Biology (Protocol number SIBCB-S330-1512).

### Cell culture

*Optn*^−/−^ MEF cells were isolated from *Optn*^−/−^mice in previous study (36, 37). *Optn*^−/−^ MEF cells and Human embryonic kidney cells (HEK293T) were maintained in Dulbecco's Modified Eagle Medium (DMEM) supplemented with 10% fetal bovine serum (FBS); All transfection experiments were performed using Lipofectamine 3000 (Thermo Fisher Scientific), following the manufacturer's instruction.

### Animal handling

Mice weighing 18–25 g (obtained from the SLRC laboratory animal center) were used for this study. The mice were randomly assigned to the PBS group, control group (lenti-virus only), OPTN group and E478G group. 2.0 μl lenti-virus or were injected into the motor cortex with a small animal stereotaxic instrument, while animals in the PBS group received vehicle. Five days after surgery, all mice were given behavioral tests for five consecutive days, brain samples were harvest after perfusion.

### Reagents and plasmids

TNF-a (Novus, NBP2-35076, USA) reconstitute with sterilized distilled water (20.0 μg/ml), and treated *Optn*^−/−^ MEF cells and 293T cells for 4 h after 1:1,000 dilution.

Anti-MAP2(Abcam, ab5392); Anti-Caspase-3(Santa cruz, sc-7148).

Plasmids used in this study as following:

All the OPTN mutants were cloned into the pCDH plasmid with a HA tag in the C terminal.

**Table:** 

**pCDH**	**pCDH-OPTN^AGins^-HA**
pCDH-OPTN-HA	pCDH-OPTN^Q398X^-HA
pCDH-OPTN^E478G^-HA	pCDH-OPTN^Q165X^-HA
pCDH-OPTN^E50K^-HA	pCDH-OPTN^Δ*UBA*^-HA
pCDH-OPTN^R96L^-HA	pCDH-OPTN^Δ*LIR*^-HA
pCDH-OPTN^Q454E^-HA	pCDH-OPTN^ΔExon5^-HA

### Dual-luciferase assay

293T and *Optn*^−/−^ MEF cells were transfected with NF-κB luciferase reporter, pRL-TK and mutants of *Optn* as indicated simultaneous. Activity of NF-κB was detected using the Dual-Luciferase® Reporter Assay System (Promega ENZO E1960) as described before (38). Briefly, after transfection, cells were detached and cell pellets were lysed in 100 μl passive lysis buffer per 24 well, dual luciferase activities were assayed separately using Varioskan LUX Multimode Microplate Reader.

### Quantitative real-time PCR

Total RNA was extracted from *Optn*^−/−^MEF cells, human 293T cell lines and mouse brain tissues using the standard Trizol method. Synthesis of cDNA was performed using GoScript™ Reverse Transcriptase according to the manufacturer's instruction (Promega, A5001), with slight modifications (39). Quantitative PCR amplifications of indicated genes were performed using GoTaq® qPCR Master Mix (Promega, A6001) on a FTC-3000 real-time PCR machine (funglyn biotech) with GAPDH as a normalization control. After the initial denaturation (2 min at 95°C), amplification was performed with 40 cycles of 15 s at 95°C and 60 s at 60°C. All qPCR data were presented as mean ± SEM. The sequences of the primers used for qPCR are listed below:

**Table:** 

**m-GAPDH**	**5′-AGGTCGGTGTGAACGGATTTG-3′**
	5′-TGTAGACCATGTAGTTGAGGTCA-3′
m-TNF -α	5′-CATCTTCTCAAAATTCGAGTGACAA-3′
	5′-TGGGAGTAGACAAGGTACAACCC-3′
m-IL-1β	5′-AAG GAG AAC CAA GCA ACG ACA AAA-3′
	5′-TGG GGA ACT CTG CAG ACT CAA ACT-3′
m-IL-6	5′-GAGGATACCACTCCCAACAGACC-3′
	5′-AAGTGCATCATCGTTGTTCATACA-3′
m-IL-10	5′-ATAACTGCACCCACTTCCCAGTC-3′
	5′-CCCAAGTAACCCTTAAAGTCCTGC-3′
h-GAPDH	5′-CTGGG GACGACATGGAGAAAA-3′
	5′-AAGGAAGGCTGGAAGAGTGC-3′
h-TNF-α	5′-TTGCTGCCACTCAGAAACAG-3′
	5′-ATCTGCCACAGTCCACCTG-3′
h-IL-1β	5′-AAGCTGATGGCCCTAAACAG-3′
	5′-AGGTGCATCGTGCACATAAG-3′
h-IL-6	5′-GGAGACTTGCCTGGTGAAAA-3′
	5′-GTCAGGGGTGGTTATTGCAT-3′
h-IL-10	5′-GGTTGCCAAGCCTTGTCTGA-3′
	5′-AGGGAGTTCACATGCGCCT-3′

### Packaging of lenti-virus

For lenti-virus production, 293T cells in 10 cm plate were transfected with 10 μg lentiviral vectors and along with Δ8.91, VSVG vectors (pCDH: Δ 8.91: VSVG = 5: 3: 2). Twenty-four hours after transfection, the viral supernatants were collected and filtered. Virus were concentrated with 1/4V pre-cold PEG8000 concentration buffer (200g/L PEG8000; 146.1 g/L NaCl) in 4°C for 4 h, then collect the pallet after 4,000 rpm for 20 min, resuspend in 100 μl PBS. Lentiviral titer was about 1 × 10^8^ TU/ml.

### Intra-motor cortex microinjection

Ten-week old C57BL/6 mice were subjected to intra-motor cortex microinjection using small animal stereotaxic instrument (Yuyan, Shanghai, China). Briefly, mice were anesthetized with 20 μl/g 3% pentobarbital sodium. After shaving mice's hair on skull, then fixed mice into the brain locator maxillary fixator, with two ear bars pushed into the external auditory canal of the mice and fix the maxillary until the head was horizontal and cannot move freely. Craniotomy was performed, after the midline incision, seeing the bregma and then positioned the syringe on the positioner to locate the bregma and then locate the motor cortex (2.0 mm below the bregma, 1.5 mm beside the sagittal suture) and mark it. Periosteum was digested with 3.0% H_2_O_2_, and then using a hand-held cranial drill with a 0.5 mm drill bit over the motor cortex to drill a hole. Using a microinjector to aspirate 2 μl of the virus and then position the syringe on the positioner and injected into the motor cortex zone in depth of 2.0 mm with a velocity of 1.0 μl per 3 min. Suture the skin and disinfection with iodophor.

### Rotarod test

Motor coordination and balance were evaluated with the accelerating-rotarod test as described before with minor modifications (40). Mice were placed on an accelerating rotarod (DXP-3, Chinese academy of medical sciences). Setting the rotarod accelerate from 5.0 to 50.0 rpm in 2 min and hold at a constant speed for further 2 min. The latency to fall was recorded. Mice were given a session and measured 3 trails per day with a 20 min interval and repeated for 5 consecutive days.

### Balance beam test

The ability of the mice to maintain balance was evaluated as previously described with minor modifications (40, 41). Mice were placed on one end of a horizontal wooden bar (0.9 × 0.9 × 50 cm) 40 cm above the ground with a cage below, a dark goal box on another end to encourage the mouse to run toward a dark and safe environment. The time taken to traverse the beam was recorded and the speed to cross the beam was calculated. Mice were given a session and measured three trails per day with a 20 min interval and repeated for 5 consecutive days.

### Footprint test

To analyse the mice's gait we used the footprint test described previously with minor modifications (40). Non-toxic waterproof paint was used to dip the mouse's paws (red color was used for forepaws and black for hind paws). Mice were placed on one end of a straight narrow tunnel (10.0 × 10.0 × 70.0 cm) with a sheet of white paper on the bottom to record the footprints. Measurement for three steps were recorded. The parameters included front base width, hind base width, forelimb stride length, hindlimb stride length, overlap between hindlimb and forelimb and the speed to cross the tunnel (Figure S3C).

### Grip strength test

Muscle tension was measured by grip strength test described previously with minor modifications (42). Briefly, grip strength was measured by recording latency of time the mouse was able to hold on a steel wire (9.0 mm in diameter, 50.0 mm in length) suspended 40.0 cm above the horizontal surface of the ground. The latency to fall off the wire was recorded over a maximum observation period of 5 min. Mice were given a session and measured three trails per day with a 20 min interval and repeated for 5 consecutive days.

### Enzyme-linked immunosorbent assay(ELISA)

Cell culture medium or brain homogenates were harvested. Briefly, tissue was weighed and homogenized in 10 volumes per weight of RIPA buffer with protease Inhibitor. Total protein concentrations were determined using a Bradford Protein Assay Kit (PQ0012, MULTZ SCIENCES). Cytokines were measured using the Mouse IL-1β Valukine ELISA Kit (VAL601, R&D, USA), Mouse IL-6 Valukine ELISA Kit (VAL604, R&D, USA), Mouse IL-10 Valukine ELISA Kit (VAL605, R&D, USA), Mouse TNF-A Valukine ELISA Kit (VAL609, R&D, USA). Measurement of the absorption was performed using a VABIOSKANLVA (Thermo, USA). A standard curve for each analyze was curvefitted, allowing determination of the cytokine concentration in pg /mL sample volume in each well of cell culture medium. While in the brain tissue which was normalized to total protein input, expressed as pg cytokine/mg total protein.

### TUNEL assay

The measurement of neuronal cell death was carried out with TUNEL Cell Apoptosis Detection Kit (Beyotime C1091, China) following the manufacturer's instruction. Briefly, paraffinized brain tissue sections were deparaffinized and rehydrated. Subsequently digesting protein with 20.0 mg/ml proteinase K for 20 min at RT. Endogenous peroxidase was inactivated with 3% H_2_O_2_ in PBS for 20 min at RT. Labeling with biotinylated dUTP in TdT enzyme buffer incubated at 37°C for 1.0 h. After stopping the enzymatic reaction, sections were washed with PBS, covered with anti-digoxigenin peroxidase conjugate and incubated for 30 min at RT in a humid chamber. Then, sections were incubated in diaminobenzidine (DAB) until color development was achieved. Finally, sections were washed, counterstained with haematoxylin, dehydrated and mounted for microscopic detection (43).

### Immunohistochemistry

After behavioral tests, mice were sacrificed for histochemical and immunohistochemically examinations. Before the following procedures, animals were deeply anesthetized with 400.0 μl 0.3% sodium pentobarbital and transcardially perfused with 30.0 ml 0.9% saline, followed by 40.0 ml 4% paraformaldehyde in 100.0 mM phosphate buffer (PB, pH 7.4, 4°C). Brains were quickly removed and post-fixed in 4% paraformaldehyde (for paraffin section) overnight at 4°C, or cryoprotected in 30% sucrose (frozen section) separately. For frozen section examination, samples embedded in optimal cutting temperature (OCT) compound and quick-freeze in liquid nitrogen. After sectioned at 4.0 μm with a Cryostat Microtome, sections incubated at 4°C for overnight with anti-GFAP antibody (1:200, BIOSS). After rinsed in 10.0 mM PBS for three times (5 min/time), the sections were applied with secondary antibodies goat-anti-rabbit IgG(Cy3) (1:2,000, abcam) at room temperature for 4.0 h, followed by three rinses (5 min/time) in 100 mM PBS, after mounting with Fluoro-Gel, pictures were taken with the LEIKA fluorescence microscope.

For hematoxylin and eosin (H&E), CD45, and Iba-1staining, brains were perfused with 1 × PBS then fixed and store in 4% paraformaldehyde. The IHC was performed following the IHC-paraffin protocol (Abcam). Tissues were dehydrated, embedded in paraffin, sectioned at 4.0 μm, de-paraffinizing and rehydrating the section; antigen retrieval; immunohistochemically staining; dehydrating and stabilizing with mounting medium; viewing the staining under the microscope. Briefly, brain sections were pre-treated with 0.3% H_2_O_2_ in 10.0 mM PBS (pH 7.4, 4°C) for 30 min. Sections were incubated at 4°C for overnight with anti-CD45 (1:400, BIOSS) antibody, or anti-Iba1 (1:1,000, abcam). After rinsed in 10.0 mM PBS for three times (5 min/time), the sections were applied with secondary antibodies anti-rabbit IgG horse radish peroxidase(HRP) (1:200, Sigma) at room temperature for 2 h, followed by three rinses (5 min/time) in 10 mM PBS. The peroxidase reaction was performed using 3, 39-diaminobenzidine (DAB, 0.05% in 10.0 mM PBS, pH 7.4, Sigma) for 2–8 min, and then the sections were mounted onto gelatin-coated slides, routinely dehydrated, cleared and covered with neutral balsam for microscopic detection. Co-immunofluorescent staining was performed with combing anti-caspase-3(1:200, abcam) and anti-MAP2(1:200, abcam) staining using the same steps.

### Immunoblotting analysis

*Optn*^−/−^ MEF cells or brain tissue extracts were prepared in the RIPA buffer with proteasome inhibitors [50.0 mM Tris-Cl, 150.0 mM NaCl, 1.0% (v/v) Triton X-100, 1.0% sodium deoxycholate, 0.1% SDS, 2.0 mM sodium pyrophosphate, 25.0 mM β-glycerolphosphate, 1.0 mM EDTA, 1.0 mM Na_3_VO_4_,and 0.5 ug/ml leupeptin, pH 7.4] for 30 min and then centrifuged at 14,000 rpm at 4°C for 15 min to remove the cell debris. Supernatants were separated by SDS-polyacrylamide gel electrophoresis(PAGE). p- IκBα, p-p65 in the lysates were checked by immunoblotting with anti-phospho-IκBα (cell signaling), anti-phospho-p65 (cell signaling).

### Statistical analysis

Data were analyzed using Prism 7 (GraphPad Software. San Diego, California, USA, http://www.graphpad.com) and presented as mean ± SEM. Shapiro-Wilk normality test was also performed to check out the distribution style. Multiple comparisons were carried out using analysis of variance (ANOVA), with one-way ANOVA test employed when only one variance was studied and two-way ANOVA employed when more than one variances were present. Dunnett's multiple comparison tests were further carried out for *post hoc* analysis to address whether there is a statistically significant difference between two groups. In all experiments, *P-*value ≤ 0.05 was considered to be statistically significant, ^****^p ≤ 0.0001, ^***^p ≤ 0.001,^**^ *p* ≤ 0.01,^*^ *p* ≤ 0.05, without asterisks means no significance *p* > 0.05. In the whisker boxplots, results were presented based on the median value and its quartiles.

## Author contributions

ZL conceptualization, supervision, formal analysis, investigation, writing original draft, and funding acquisition. HL, CH, TY, and MC formal analysis and investigation. CC and ZW investigation. QY and CL resources. QW conceptualization and writing review and editing. HX conceptualization, investigation, and writing review and editing. RH conceptualization, resources, funding acquisition, validation, and writing review and editing.

### Conflict of interest statement

The authors declare that the research was conducted in the absence of any commercial or financial relationships that could be construed as a potential conflict of interest.

## Supplementary material

Figure S1 Related to Figure 1. OPTN^E478G^ abolished NF-κB activation-inhibiting effect in 293T cells. **(A)**. NF-κB activity was measured by dual luciferase reporter assay after transfecting 293T cells with NF-κB reporter and the mutants of *OPTN* plasmid as indicated. Data were presented in mean ± SEM, *n* = 4. Data analysis was performed by comparing each group to OPTN group using one way ANOVA and Dunnett's multiple comparison test, ^****^*p* ≤ 0.0001, without asterisks means no significance *p* > 0.05. **(B)**. NF-κB activity was measured by dual luciferase reporter assay after transfecting 293T cells with NF-κB reporter and the mutants of *OPTN* plasmid as indicated. 24 h after transfection, cells were treated with 20 ng/ml TNF-a for 4 h before harvest. Data were presented in mean ± SEM, *n* = 4. Data analysis was performed by comparing each group to OPTN group using one way ANOVA and Dunnett's multiple comparison test, ^****^*p* ≤ 0.0001, ^***^*p* ≤ 0.001, ^*^*p* ≤ 0.05, without asterisks means no significance *p* > 0.05.

Figure S2 Related to Figure 2. Secretion of cytokines was analyzed in 293T cells transfected with ALS-associated mutants. **(A)**. Cytokines (IL-1β, IL-6, IL-10, TNF-a) in the supernatants of the cell culture medium were analyzed by enzyme-linked immunosorbent assay (ELISA). Data were presented as mean ± SEM, *n* = 3. Data analysis was performed by comparing each group to OPTN group using one way ANOVA and Dunnett's multiple comparison test, ^****^*p* ≤ 0.0001, ^***^*p* ≤ 0.001,^**^*p* ≤ 0.01,^*^*p* ≤ 0.05, without asterisks means no significance *p* > 0.05. **(B)**. Cytokines (IL-1β, IL-6, IL-10, TNF-a) were measured by quantitative real-time PCR. Data were presented as mean ± SEM, *n* = 3. Data analysis was performed by comparing each group to OPTN group using one way ANOVA and Dunnett's multiple comparison test, ^****^*p* ≤ 0.0001, ^***^*p* ≤ 0.001,^**^*p* ≤ 0.01,^*^*p* ≤ 0.05, without asterisks means no significance *p* > 0.05.

Figure S3 Related to Figure 4. Infection of OPTN^E478G^ virus increased nerve cell death and impaired motility of the mice. **(A)**. Quantification of CD45-positive cells. Values were presented as mean ± SEM, *n* = 6. Data analysis was performed by comparing E478G group to OPTN group using unpaired t test, ^****^*p* < 0.0001. **(B)**. Quantification of Iba-1-positive cells. Values were presented as mean ± SEM, *n* = 6. Data analysis was performed by comparing E478G group to OPTN group using unpaired t test, ^****^*p* < 0.0001. **(C)**. Schematic representation to show the footprint test. FW, front base width; HW, hind base width; FL, forelimb stride length; HL, hindlimb stride length; OV, overlap between hindlimb and forelimb.
