# Supplementary figures and images for: ALS-Associated E478G Mutation in Human OPTN (Optineurin) Promotes Inflammation and Induces Neuronal Cell Death
Source: Front Immunol. 2018 Nov 14;9:2647. doi: 10.3389/fimmu.2018.02647 (PMC6251386; doi:10.3389/fimmu.2018.02647)

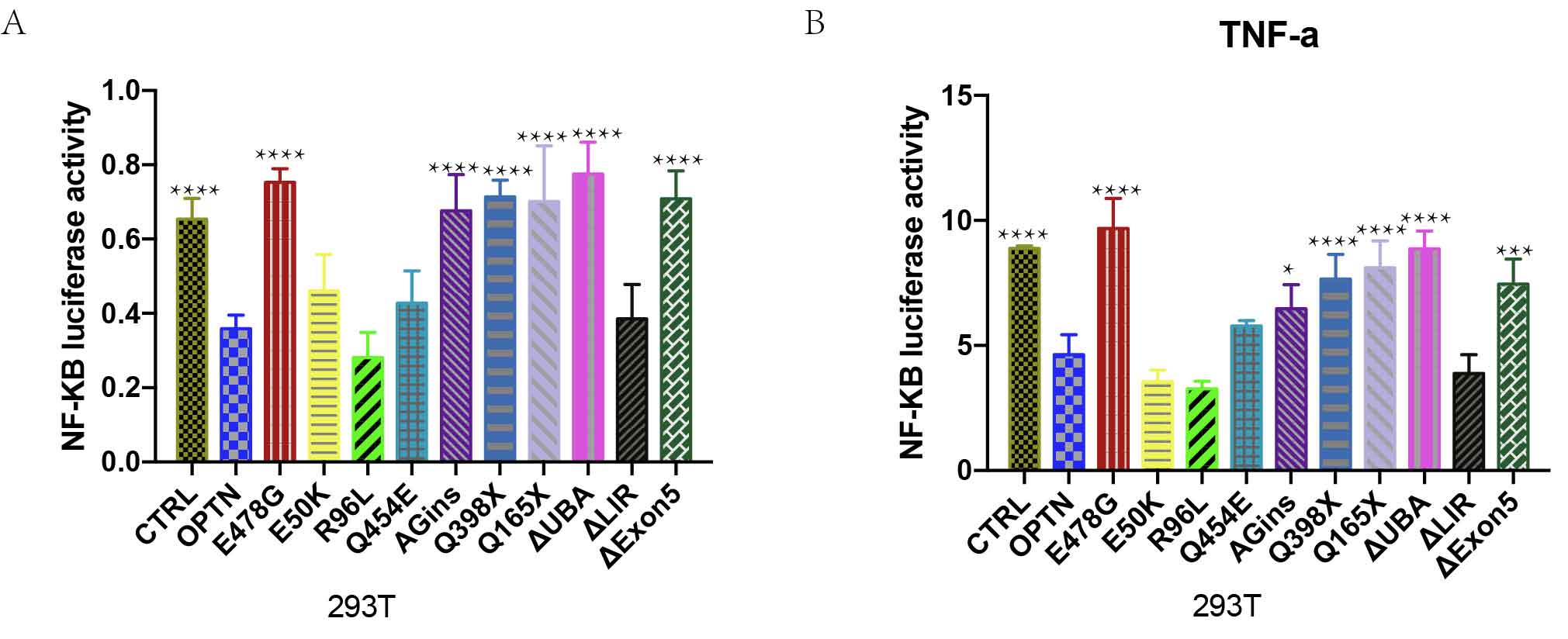

Supplement: Figure S1 — Related to Figure 1. OPTNE478G abolished NF-κB activation-inhibiting effect in 293T cells. (A). NF-κB activity was measured by dual luciferase reporter assay after transfecting 293T cells with NF-κB reporter and the mutants of OPTN plasmid as indicated. Data were presented in mean ± SEM, n = 4. Data analysis was performed by comparing each group to OPTN group using one way ANOVA and Dunnett's multiple comparison test, ****p ≤ 0.0001, without asterisks means no significance p > 0.05. (B). NF-κB activity was measured by dual luciferase reporter assay after transfecting 293T cells with NF-κB reporter and the mutants of OPTN plasmid as indicated. 24 h after transfection, cells were treated with 20 ng/ml TNF-a for 4 h before harvest. Data were presented in mean ± SEM, n = 4. Data analysis was performed by comparing each group to OPTN group using one way ANOVA and Dunnett's multiple comparison test, ****p ≤ 0.0001, ***p ≤ 0.001, *p ≤ 0.05, without asterisks means no significance p > 0.05. [file Image_1.JPEG]

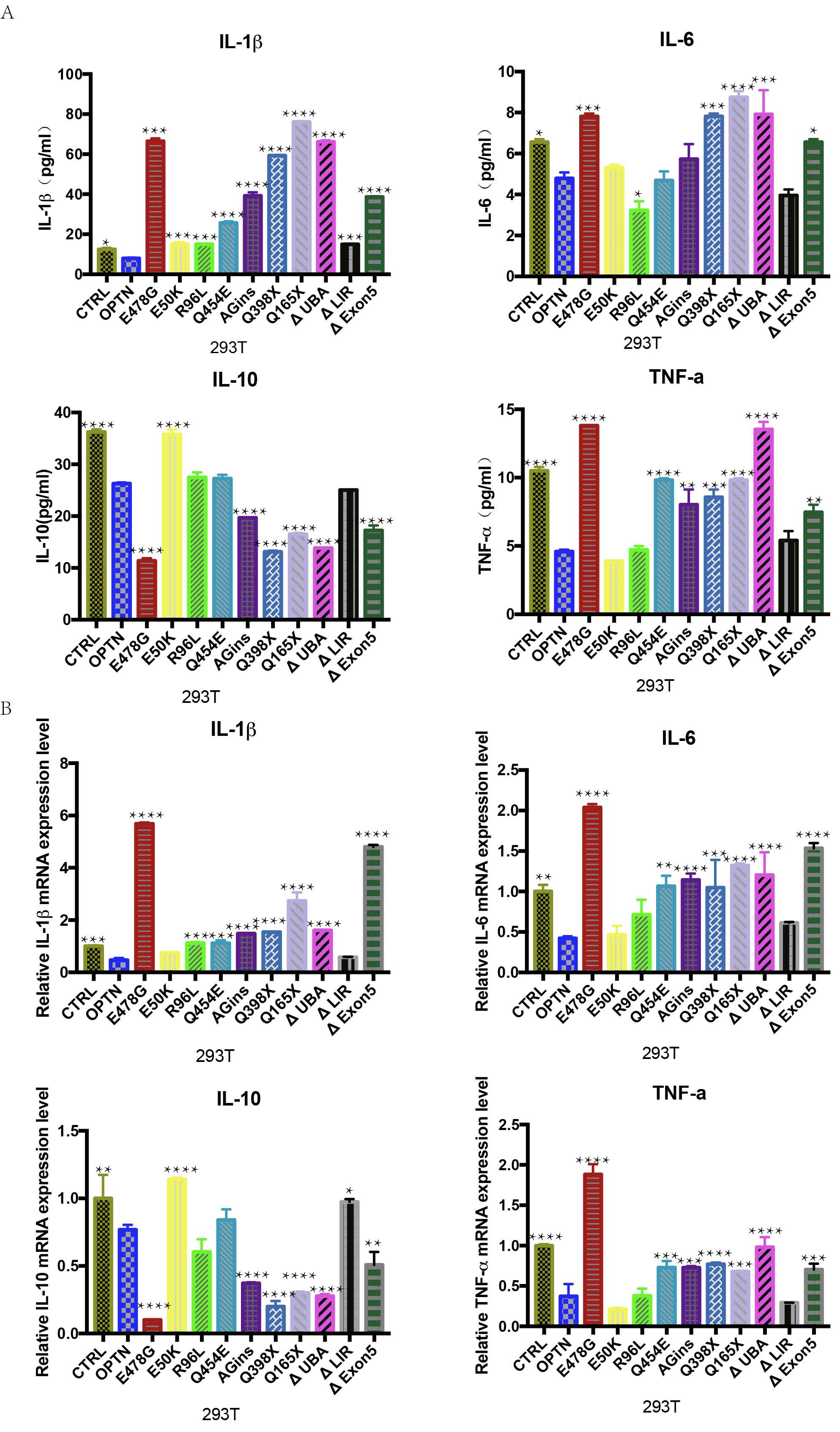

Supplement: Figure S2 — Related to Figure 2. Secretion of cytokines was analyzed in 293T cells transfected with ALS-associated mutants. (A). Cytokines (IL-1β, IL-6, IL-10, TNF-a) in the supernatants of the cell culture medium were analyzed by enzyme-linked immunosorbent assay (ELISA). Data were presented as mean ± SEM, n = 3. Data analysis was performed by comparing each group to OPTN group using one way ANOVA and Dunnett's multiple comparison test, ****p ≤ 0.0001, ***p ≤ 0.001,**p ≤ 0.01,*p ≤ 0.05, without asterisks means no significance p > 0.05. (B). Cytokines (IL-1β, IL-6, IL-10, TNF-a) were measured by quantitative real-time PCR. Data were presented as mean ± SEM, n = 3. Data analysis was performed by comparing each group to OPTN group using one way ANOVA and Dunnett's multiple comparison test, ****p ≤ 0.0001, ***p ≤ 0.001,**p ≤ 0.01,*p ≤ 0.05, without asterisks means no significance p > 0.05. [file Image_2.JPEG]

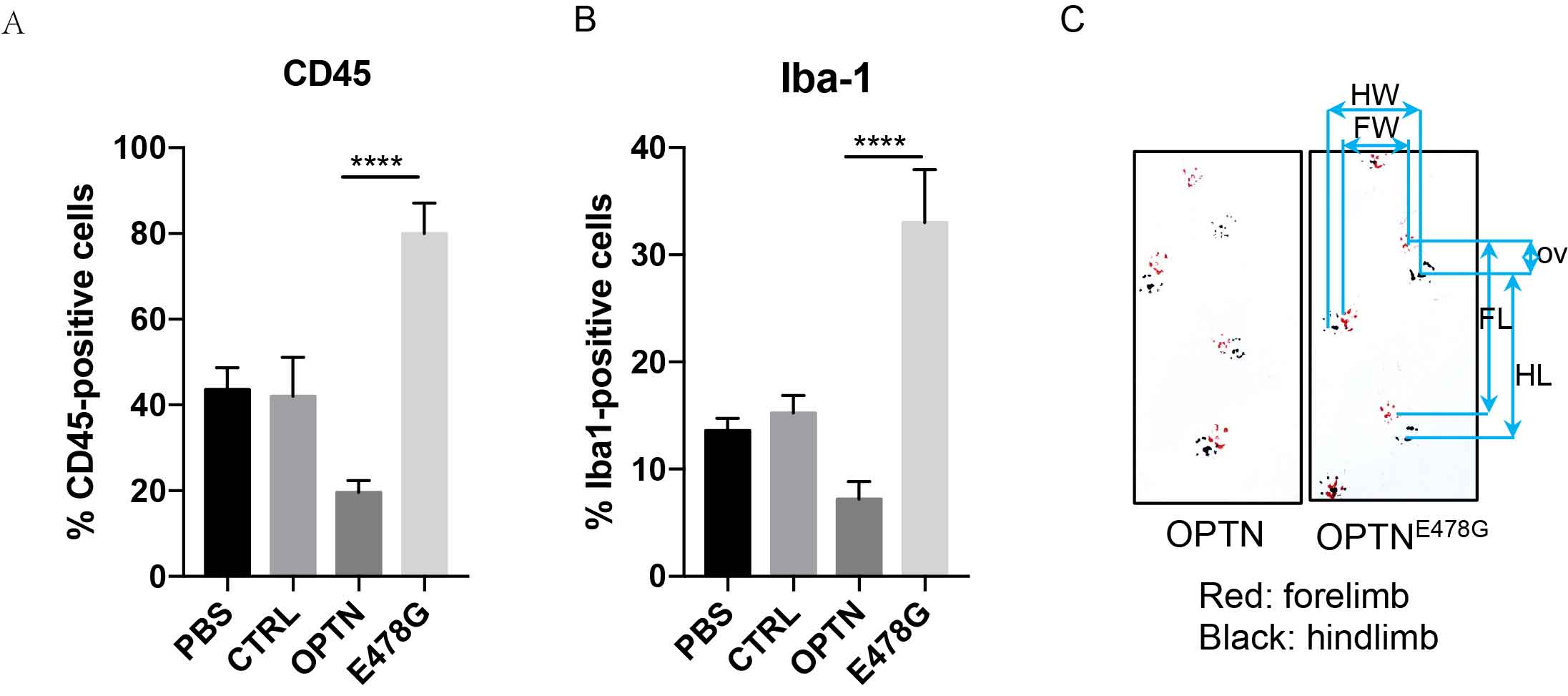

Supplement: Figure S3 — Related to Figure 4. Infection of OPTNE478G virus increased nerve cell death and impaired motility of the mice. (A). Quantification of CD45-positive cells. Values were presented as mean ± SEM, n = 6. Data analysis was performed by comparing E478G group to OPTN group using unpaired t test, ****p < 0.0001. (B). Quantification of Iba-1-positive cells. Values were presented as mean ± SEM, n = 6. Data analysis was performed by comparing E478G group to OPTN group using unpaired t test, ****p < 0.0001. (C). Schematic representation to show the footprint test. FW, front base width; HW, hind base width; FL, forelimb stride length; HL, hindlimb stride length; OV, overlap between hindlimb and forelimb. [file Image_3.JPEG]
